# Relationship of the Use of Short Footwear with the Development of Hallux Valgus in a Sample of Andalusian Schoolchildren

**DOI:** 10.3390/ijerph182111244

**Published:** 2021-10-26

**Authors:** María Luisa González-Elena, Aurora Castro-Méndez, Manuel Coheña-Jiménez, Antonio Córdoba-Fernández

**Affiliations:** Department of Podiatry, Faculty of Nursing, Physiotherapy and Podiatry, University of Sevilla, 41009 Sevilla, Spain; maruchi1@us.es (M.L.G.-E.); auroracastro@us.es (A.C.-M.); mcohena@us.es (M.C.-J.)

**Keywords:** footwear fit, hallux valgus, feet deformities, indoor shoes, schoolchildren, functional length excess

## Abstract

Background: Several studies have shown the relationship between poor footwear fit and the risk of feet deformities. The available evidence seems to show that the development of hallux valgus deformity in the feet of schoolchildren may be related to the use of shoes that are poorly fitting in length. The objective of this cross-sectional study was to analyze the relationship between poor footwear fit in length and risk of developing hallux valgus. Methods: Using an instrument that was designed and calibrated for this purpose, maximum foot length was obtained and compared to the inner length of the shoe in 187 schoolchildren. Hallux valgus angle (HVA) was measured on weight-bearing podogram image obtained from the longest foot in 188 schoolchildren. Results: By default, the footwear was poorly fitting in length (too short or close-fitting) in 38.5% of the schoolchildren, with boys having the worst footwear fit; though no significant differences stood out. (*p* = 0.276). Regarding the HVA, no significant differences were recorded according to age or gender (*p* = 0.573). A strong correlation was observed between too-short footwear and the increase in HVA in 10-year-old boys (*r* = 0.817; *p* = 0.025) and in 9-year-old girls (*r* = 0.705; *p* = 0.005). Conclusions: Inadequate footwear fit in length may be a predisposing extrinsic risk factor for the development of hallux valgus in schoolchildren of both sexes. Results of the present study demonstrate the need to adapt the sizes of footwear to the rapid increase in foot-length that occur at puberty to avoid the risk of developing hallux valgus, especially at the ages of onset pubertal foot growth.

## 1. Introduction

Several studies have analyzed the relationship between poor footwear fit and risk of foot deformities, and the available evidence shows that a large proportion of the population wears incorrectly-sized footwear, which is associated with foot pain and foot disorders [1]. The influence of footwear on the appearance of foot deformities seems to have a notable impact at an early age since the maturation of the bone structure of children’s feet has not yet finished. Recent studies carried out on schoolchildren show that between 4 and 12 years of age, there is an important progressive growth especially in length in the feet of children [2,3]. These rapid changes may be conditioned by extrinsic factors, such as footwear, or intrinsic factors, such as gender, foot morphology or body mass index, among others, which in combination can influence the morphological and functional development of the adult foot [3,4]. Based on the observations made in different studies on the high rate of ill-fitting footwear in schoolchildren, several investigations highlight the importance of making frequent changes in footwear, especially before Tanner’s stage in boys and premenarchal girls to prevent the risk of developing deformities in the feet early on [3,4,5,6,7,8,9].

Since the etiology of juvenile hallux valgus is multifactorial, extrinsic factors such as the use of ill-fitting footwear seem to be related to the development of the deformity. Some studies show that the deformity is more prevalent in populations that wear footwear and that the hallux valgus angle (HVA) in children who go barefoot is significantly smaller compared to those who habitually wore indoor shoes. According to previous studies, specific problems (pain, hallux valgus, claw toes or skin changes) have been detected in the feet of children who used inappropriate footwear) [10,11]. Similarly, the use of ill-fitting footwear seems to be related to the development of hallux valgus in schoolchildren [9,10,11,12,13,14,15]. This current study aims to evaluate the influence that an inadequate footwear fit in schoolchildren can have on the development of the deformity and establish if there is a correlation between the use of poorly fitting outdoor footwear and the development of hallux valgus in a sample of schoolchildren from southern Spain. 

## 2. Materials and Methods

### 2.1. Participants

This cross-sectional study was conducted between February and May 2019, following the established criteria of Strengthening the Reporting of Observational Studies in Epidemiology (STROBE) and it was approved by the Research Ethics Committee of the University of Seville (ref. 0711-2013). Through purposive sampling and using as the selection criterion the geographical proximity of participants to the Faculty of Nursing, Physiotherapy and Podiatry of the University of Seville the study was carried out. Two centers from the city of Seville (Spain) were selected. The randomized sample included one Primary Education center and one Secondary center. For the sample selection, a probabilistic sampling was used from a study population composed of 230 schoolchildren. With the consent of their parents or guardians, schoolchildren between 3 and 15 years of age were selected, who did not have any foot disorders or malformations (pes adductus, visible bunion deformities) or previous history of foot surgery. The children who wore boots or high-top footwear on the day of examination were excluded from the sample. The final study sample consisted of 188 schoolchildren.

### 2.2. Data Collection

A calibrated measuring instrument was used to determine the maximum length and width in the longest foot of schoolchildren. When the measurement obtained was identical for both feet, it was randomly selected by tossing a coin into the air (head = left foot, tail = right foot).With an extendable tape measure placed from the heel to longest toe, foot length was measured with the student in a weight-bearing position, with his feet at the same height and with his knees stretched out on an acetate placed on a methacrylate surface with a printed calibrated template. The length of the foot was obtained directly by a transparency sheet through the grid scale and the width was obtained from the X and Y 115 coordinates of each point using Access 11.0 (Microsoft Office 2010) software. Maximum length in millimeters of the foot was considered as the distance from the back of the heel to the longest toe, and the maximum width as the distance in millimeters from the most prominent medial and lateral points corresponding to the heads of the first and fifth metatarsals. Three different digital formulas were considered; Egyptian foot (first longest toe), Greek foot (second longest toe) or square foot (first and second toe of the same length). Once these measurements were obtained, they were transferred to the inside of the footwear using acetate transparent with the same size as the child’s foot where the length and width reference points obtained were placed. Subsequently telescopic gauges with protactors were used to check the footwear. The gauge had a brake that could be released once inside of shoe, which allowed to edit the fit to expand longitudinally and transversally. Once the gauge was extracted from the shoe, length and width were calculated using a retractable measuring tape. All the measuring instruments were designed and evaluated by the Andalusian Centre of Metrology and have been used in previous studies [3,5].

**Figure 1:**
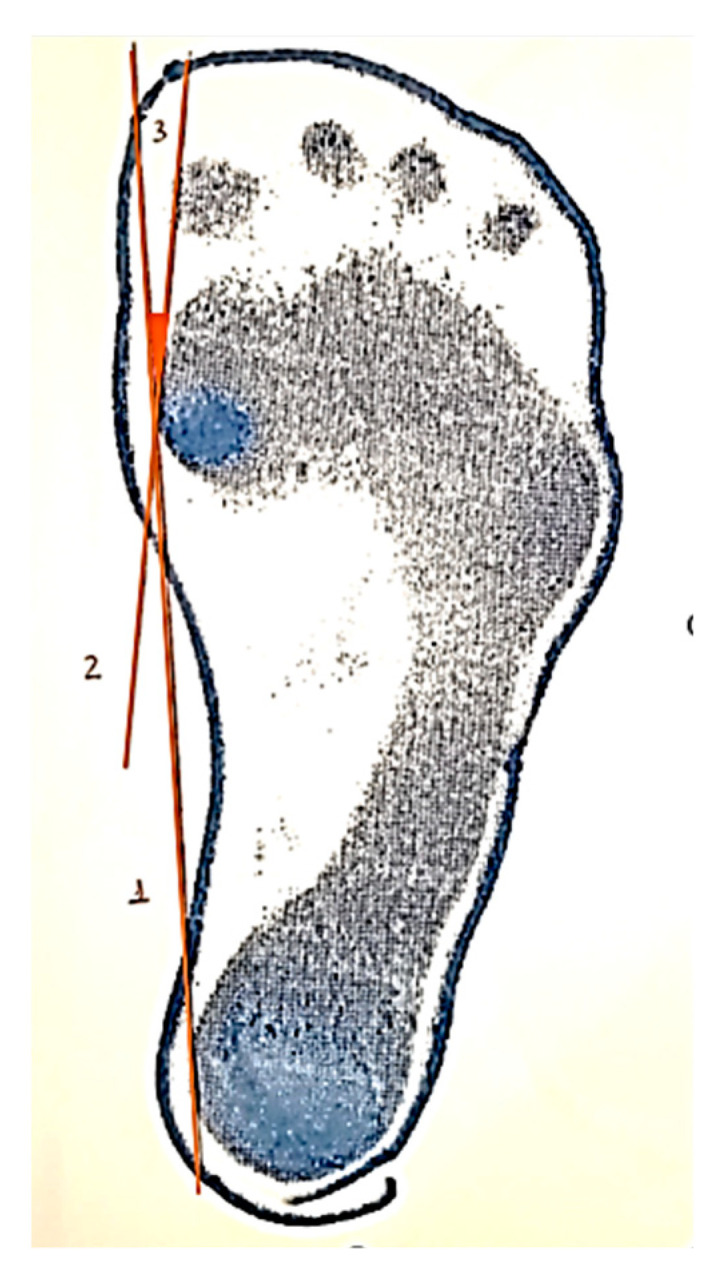
Measure method to evaluate the hallux valgus angle.

HVA in studied feet were measured using a podogram taken with the student in a relaxed position with the arms along the body, and body weight distributed evenly on both feet. HVA was considered as the angle formed by intersection of the line between the most medial point of the first toe and the most medial point of the metatarsal head, and another line between the most medial edge of the heel and the most medial point of the first metatarsal head (Figure 1).

The inter-observer reliability of the researchers and the measurements were calculated in an interval of 1 week. Inter-observer intraclass correlation coefficient (ICC) with level of confidence of 95% (Cronbach’s α) was 0.995 (CI = 0.993–0.997) for HVA. Coefficient of variation (CV) was 1.7% and inter-observer relative technical error of measurement (TEM %) was 0.20. All measurements were reported by only one independent observer. Based on what was described by other authors, four categories were established in terms of length fit of footwear; short shoe (Shoe  ≤  than foot to 1–5 mm longer than foot), close-fitting shoe (shoe 6–9 mm longer than foot), correct fit (10–15 mm space in shoe) and too-long shoe (16mm or more space in shoe) [4,7,9,10].

### 2.3. Sample Size Calculation

The size of sample was calculated for a power of 0.95, with alpha error of 0.05 and a size effect of 0.25 (G*Power 3.1.9.4, Franz Faul, University of Kiel, Kiel, Germany). This calculation produced a necessary sample size of 140 subjects.

### 2.4. Statistical Analysis

To compare the length adjustment, the Wilcoxon signed range test was used for related samples. To compare the length adjustment as a function of gender, the Mann–Whitney U test was used. To analyze the relationship between the fit and the HVA, the Kruskal–Wallis test was used for independent samples. The comparison between the groups according to gender were done via the Mann–Whitney U test. A 95% confidence interval was considered for inferential analysis; therefore, the experimental *p*-value was compared with a significance level of 5%. Data were analyzed with the IBM^®^ SPSS Statistics statistical program version 25 for Windows 10 (IBM Corp., Armonk, New York, NY, USA).

## 3. Results

The final size of the cohort was 187 schoolchildren, of whom 90 were boys and 97 girls, with an average age of 8.07 ± 2.93 years (mean ± SD, median, interquartile range, Z and *p* value). The basic descriptive statistics of the bodily characteristics of the examined children and distribution according to age group and gender are shown in Table 1 and Table 2. 

Regarding the length of the foot according to age and sex, there were no significant differences (*p* = 0.518). Except for the 4-year age group where the children wore significantly longer footwear (*p* = 0.047), there were no significant differences between the average inner length of the footwear and the average length of the foot according to gender. There were also no significant differences in the interior length of footwear depending on age and sex (*p* = 0.324). Significant differences were recorded between the average values in shoe length and foot length. In relation to the fit of the footwear and according to the preset fitting categories, footwear was poorly fitting (too short or with insufficient length) in 42.7% of boys and 35.1% of girls. The age range where the worst fit occurred by default was at age 9 in girls and at age 10 in boys. (Table 3 and Table 4). 

Regarding the type of digital formula observed, the most prevalent forefoot morphotypes corresponded to the formulas of Greek foot and Egyptian foot although without significant differences between any of the categories or for any of the groups according to gender (Table 5).

The mean value of HVA recorded was higher in girls than boys but without significant differences (6.91 ± 4.61 vs. 6.47 ± 3.88; *p* = 0.697). The highest HVA mean value recorded corresponded to the footwear category considered short. There were no significant differences between the average value of HVA based on the digital formula presented for any of the groups (Table 3 and Table 4). There were also no significant differences between the average HVA value between the categories too-short and close-fit shoe and correct shoe and too-long shoe (Table 6 and Table 7).

**Figure 2:**
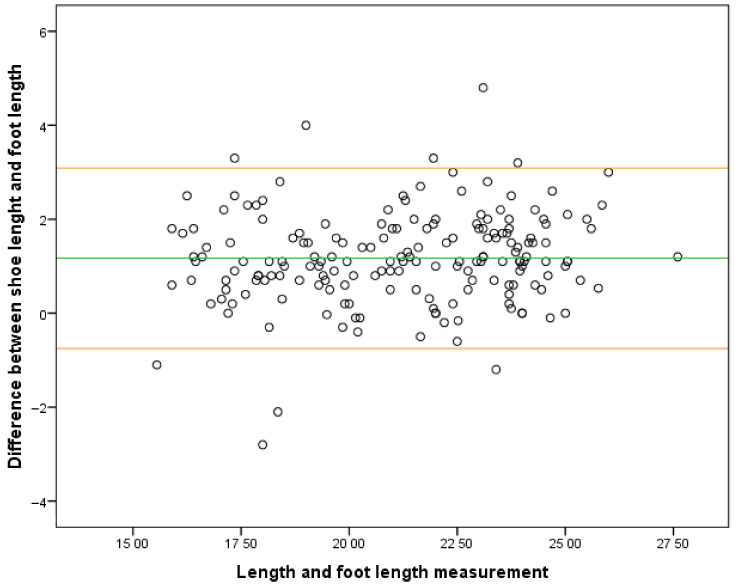
Bland–Almant plot.

In categories where footwear was poorly fitting in length, a strong correlation was observed between too-short footwear and an increase in HVA in 10-year-old boys (*r* = 0.817; *p* = 0.025) and in 9-year-old girls (*r* = 0.705; *p* = 0.005). The Bland–Almant plot shows the differences between measurements (Figure 2).

## 4. Discussion

The issue of poorly fitting indoor shoes is often overlooked. However, wearing shoes of insufficient length during childhood has often been cited as leading to deformities of the foot [9,10,14]. We found that 38.5% of the outdoor shoes were too short in length, that is, the difference between the length of the foot and the inside length of the shoe was less than 9 mm. This percentage, although relevant, is considerably lower than that reported in similar studies. Kinz et al. found that 75.5% of the outdoor shoes were too short in length, that is, the difference between the length of the foot and the inside length of the shoe was less than 10 mm [10]. These shoes did not allow the children’s feet the extra space that is recommended for healthy podiatric development and function. In the present study a 38.5% of the outdoor shoes tested were even shorter or only close-fitting (as long as the child’s foot), exposing the foot to permanent strain. This percentage is similar to that reported in similar studies carried out in European schoolchildren [14]. However, the footwear fit in the children in our sample was considerably better than reported in other European preschoolers and elementary school-aged children studies [5,9,10].

In our study we have detected worse footwear fit in the boys without significant differences. These data are not consistent with other studies carried out in schoolchildren in our country where it was observed that girls from 8 to 9 years old had a significantly worse footwear fit in length than boys [6]. Recent observational studies carried out in schoolchildren in southern Spain have shown that the age of onset pubertal foot growth was 7–8 years in girls and 8–9 years in boys and that, in the age range between 7 and 11 years, an adequate footwear fit in length is essential [3,5].

In the average HVA of the children’s feet depending on the fit of the individual shoe in categories where footwear was poorly fitting in length, a strong correlation was observed between too-short footwear and an increase in HVA in boys at 10 years old and in girls at 9 years old. The children with too-short shoes and close-fitting shoes had a higher average of HVA of three and two degrees of HVA regarding to correct-fitting and too-long shoes, respectively. However, unlike other studies the observed relationship between the fit length of the shoes and HVA was not significant [9,10,14]. 

Like Klein et al. we have detected a worse fit in shoe length in the boys of our sample, however contrary to the results reported by these authors, our results show a slightly higher average of HVA in the girls although without significant differences [9]. As in previous studies, we have observed a strong correlation between poorly fitting footwear and increased HVA in schoolchildren of both sexes in ages of onset pubertal foot growth, especially in girls [9,13]. The highest average value of HVA was recorded in the footwear category considered short. These data are similar to reported in previous studies [10] and markedly higher than those observed in other investigations [15]. These differences can be attributed to different methods of measuring the HVA, since the first employed the method described by Klein et al. obtained by drawing two tangents on the margo medialis pedis, while the second employed a measurement method similar to that used in this study with an electronic podoscope.

The results of our study show that girls had on average more pronounced HVA than boys although without significant differences, and that in general, older children had more pronounced angles than younger ones. This finding is in disagreement with what was observed in previous studies [9,10,14]. In these studies, the relative risk for a lateral hallux deviation when wearing shoes of insufficient length was significantly higher for boys than for girls. A recent study conducted on a sample of 100 Polish girls aged 9 demonstrated that 40% of schoolchildren are wearing shoes that are too short in length; a slightly higher percentage than that registered by us in the group of girls (35%). In this same study performed with a similar method of measurement using an electronic podoscope, the average value of HVA recorded in 9-year-old girls was 5.57 ± 4.09, very close to the one recorded in our sample (5.93 ± 2.78) [15]. It is likely that in our study this small difference in terms of gender may have been conditioned by the characteristics of outdoor footwear; possibly due to fashion trends and the use of more pointy-toed shoes. However, taking into account the multicausal nature of the deformity, we consider that these differences may also be due to the coexistence of other intrinsic factors such as heredity, pes valgus, metatarsus primus varus, etc. that were not taken into account in the present study. We have not found significant differences regarding footwear fit in length with respect to digital formula. The morphology of the forefoot has been associated with the risk of developing hallux valgus. In a case-control study carried out in older people, a high prevalence of square feet was observed in the women control group with hallux valgus and a relationship was detected between the presence of hallux valgus and its prevalence in participants with square feet, concurrent with the use of constrictive footwear. The study concludes that footwear characteristics can be considered risk factors in the alteration of the digital formula and aggravation of the deformity suffered in older women [16]. In an observational study conducted in adolescents from 12 to 18 years of age with considerably higher frequency of Egyptian foot incidence, it was observed that the valgus position of the hallux in girls was dominant [17]. In our sample, there were no significant differences in the prevalence of any of the morphotypes nor in terms of gender. Previous studies show that footwear manufacturers do not take into account the anthropometric differences related to the digital formula. Current shoe designs do not take into account the integral three-dimensional shape of the foot and do not reproduce the wide variability in forefoot morphology. Some authors report that most shoe manufacturers do not modify the last size to include feet with different digital formulas [17,18]. We also consider that when designing ergonomic footwear for schoolchildren, not only the different digital formulas should be taken into account, but also the position and orientation of the forefoot (metatarsal adduct) inside the shoe.

Our study showed a strong correlation between inadequate footwear fit and increased HVA in boys at age 10 and girls at age 9. In general, an increase in HVA with age was also observed in both sexes, especially in girls in the age group between 11 and 14 years. We believe that this circumstance may be influenced in part by the increase in foot length and width that precedes the onset of Tanner’s stage II in both sexes and that matches with the peak of longitudinal growth of the foot taking place prior to the peak of growth in height [2,3].

The data of this study are consistent with those reported in other studies carried out in schoolchildren in our country and other European countries. All of them highlight the need to establish a better fit in length of footwear for schoolchildren, especially between 6 and 13 years of age for both sexes. All of these studies conclude that during these ages, footwear must be replaced periodically. 

Bearing in mind the design of our study and the multicausality of the deformity analyzed, it is logical to consider the presence of some limitations. The main limitation of our study stems from the fact that the influence of individual heredity history was not taken into account. Some studies have shown a high heritability of foot disorders, especially hallux valgus deformity in white men and women of European descent [19]. Another limitation of our study may have been a measurement bias, a single investigator performed the evaluation of HVA before and after 1 week. Other intrinsic risk factors related to increased HVA (height, weight and BMI) were only partially investigated. Futures studies should also analyze the influence of other risk factors such as hindfoot posture. The theory is advanced that the collapse of the arch with vertical orientation (inclination) of the first metatarsal axis may be related to the deformity [20]. We did not analyze the footwear in width taking into account the lack of a stop for the touch probe due to non-resistance, typical of the characteristics of the footwear’s material in the metatarsal area. We consider that this measurement would not have been reliable.

## 5. Conclusions

Although definitive conclusions regarding causality cannot be made from cross-sectional studies, this study highlights footwear as an important factor to monitor in hallux valgus prevention in schoolchildren. It could be proven that the risk of having increased HVA in schoolchildren is related to wearing shoes of insufficient length. The fact that 38.5% of the children examined wear indoor shoes of insufficient length, points to the general health relevance of this problem. For a better understanding of the problem that will allow to establish the cause-and-effect relationships regarding the impact of wearing specific types of footwear, future prospective and longitudinal studies should be planned and extended to other regions and countries also covering other age categories.

## Figures and Tables

**Table 1:** Comparison of select bodily characteristics in respective groups of subjects.

		Mean	Standard Deviation	Median	Interquartile Range	Z	*p*
**Weight (Kg)**	**Boys** **Girls**	27.2 28.3	10.4 11.0	25 26	19–35 20–34	1.156	0.248
**Height (cm)**	**Boys** **Girls**	122.8 124.4	17.0 16.1	121 124	108–136 112–136	1.137	0.255
**BMI**	**Boys**	17.5	2.8	17	16–19	0.949	0.343
	**Girls**	17.8	3.0	17	16–19		
**Foot length (mm)**	**Boys** **Girls**	20.6 20.7	2.5 2.9	20.4 21.7	18.7–22.4 17.8–23.3	0.646	0.518
**Foot width (cm)**	**Boys** **Girls**	7.7 7.7	1.0 1.0	7.7 7.8	7.0–8.3 7.0–8.6	0.009	0.993

Z: value of Mann–Whitney U test statistic; *p*: probability value.

**Table 2:** Physical characteristics of the participants. Mean and standard deviation of foot anthropometric variables and footwear length for different age groups.

**Age**	**Males** **(N)**	**Foot** **Length** **(mm)**	**Footwear** **Length** **(mm)**	**Fit Difference**	**HVA**
3	3	174.67 ± 0.45	186.33 ± 0.75	11.6 ± 0.40	6.67 ± 3.05
4	11	169.00 ± 0.90	179.27 ± 0.74	10.27 ± 0.83	7.18 ± 3.81
5	4	183.00 ± 1.92	190.50 ± 1.89	7.50 ± 0.52	5.00 ± 4.76
6	18	193.72 ± 0.91	202.06 ± 0.89	8.33 ± 0.63	5.72 ± 3.99
7	10	202.00 ± 0.93	207.60 ± 1.79	5.60 ± 1.39	7.30 ± 3.19
8	10	217.44 ± 0.97	225.04 ± 0.92	8.15 ± 0.66	7.00 ± 2.49
9	8	212.38 ± 1.01	226.75 ± 1.26	14.37 ± 0.89	5.38 ± 5.29
10	7	224.29 ± 1.30	234.71 ± 0.84	10.42 ± 1.06	7.68 ± 5.64
11	5	218.20 ± 1.91	239.80 ± 1.27	21.60 ± 1.81	5.40 ± 4.66
12	12	243.00 ± 1.30	256.44 ± 1.74	13.42 ± 0.89	6.67 ± 3.49
13	2	223.50 ± 2.33	233.00 ± 1.83	9.5 ± 0.49	6.00 ± 4.24
**Age**	**Females** **(N)**	**Foot** **Length** **(mm)**	**Footwear** **Length** **(mm)**	**Fit Difference**	**HVA**
3	3	159.00 ± 0.90	171.00 ± 0.36	12.00 ± 0.55	5.33 ± 2.51
4	15	164.43 ± 0.86	182.47 ± 1.05	17.85 ± 0.94	8.33 ± 5.90
5	2	177.00 ± 0.90	184.00 ± 0.00	7.00 ± 0.80	6.00 ± 0.00
6	12	178.09 ± 1.30	185.06 ± 1.69	6.33 ± 1.56	7.50 ± 6.69
7	5	195.00 ± 0.65	209.00 ± 0.96	14.00 ± 0.82	6.80 ± 3.70
8	10	204.70 ± 1.46	214.50 ± 1.55	9.80 ± 0.72	4.90 ± 2.88
9	14	223.93 ± 1.11	236.14 ± 0.96	12.21 ± 1.08	5.93 ± 2.78
10	8	226.00 ± 0.77	239.75 ± 0.75	13.75 ± 0.83	4.75 ± 3.61
11	2	242.00 ± 1.13	251.00 ± 0.84	9.00 ± 0.28	7.00 ± 5.65
12	23	232.05 ± 0.91	247.14 ± 0.71	15.09 ± 0.77	8.09 ± 4.80
13	1	235.00 ± 0.63	244.00 ± 1.47	9.00 ± 1.20	10.00 ± 5.01
14	2	242.50 ± 0.35	252.50 ± 1.76	10.00 ± 1.41	8.00 ± 5.65

**Table 3:** Comparison of the medians between foot length and footwear length.

Length (mm)	Foot Length	Footwear Length	*p* Value
**Median (minimum/maximum)**	207 (150/270)	220 (150/282)	
**Mean ± SD**	206.2 ± 27.1	218.1 ± 28.5	
**Typical mean error**	2	2.1	˂0.001
**95% CI (Lower limit, Upper limit)**	202.3–210.2	213.9–222.2	

**Table 4:** Distribution according to sample length fit.

Shoe Fit Length	Total	Boy	Girl				
**Too short**	40	21.7	22	24.7	18	19.1	
**Close-fit**	31	16.8	16	18	15	16	0.276
**Correct**	52	28.3	28	31.5	24	25.5	
**Too long**	61	33.2	23	25.8	37	39.4	

**Table 5:** Distribution of the sample according to the digital formula presented.

		Total		Boy		Girl		
		N	%	N	%	N	%	*p*-Value
	Egyptian	65	34.9	30	33.7	35	36.5	
**Digital Formula**	Greek	65	34.9	29	32.6	35	36.5	0.616
	Square	56	30.1	30	33.7	26	27.1	

**Table 6:** HVA according to the type of digital formula.

		Mean	Standard Deviation	Median	Interquartile Range	Z	*p*-Value
	**Egyptian**	6.5	4.7	5	3–9		
**HVA**	**Greek**	7.2	4.5	8	4–10	1.808	0.405
	**Square**	6.4	3.5	7	3.3–9.8		

**Table 7:** Comparison between fit and HVA angle.

Too-Short Shoe	HVA	*p*-Value
**Median (minimum/maximum)**	8(0–26)	
**Mean** **± SD**	7.6 ± 4.8	
**Typical mean error**	0.8	
**95% CI (Lower limit, Upper limit**	6.1–9.1	
**I** **nsufficient length (Close-fit shoe)**		
**Median (minimum/maximum)**	7 (0–19)	
**Mean** **± SD**	7.1 ± 3.9	
**Typical mean error**	0.5	
**95% CI (Lower limit, Upper limit)**	6.1–8.2	
**Correct shoe**		0.263
**Median (minimum/maximum)**	5 (0–15)	
**Mean** **± SD**	6.0 ± 4.2	
**Typical mean error**	0.7	
**95% CI (Lower limit, Upper limit)**	4.5–7.6	
**Too-long shoe**		
**Median (minimum/maximum)**	5(0–17)	
**Mean** **± SD**	6.3 ± 4.4	
**Typical mean error**	0.6	
**95% CI (Lower limit, Upper limit)**	5.2–7.5	
	8	

*p*-Value: Difference in HVA between the categories short shoe and close-fit shoe and correct shoe and too-long shoe.

## Data Availability

Please contact acordoba@us.es with any data requests.
